# Correction: Bimetallic nanocatalysts supported on graphitic carbon nitride for sustainable energy development: the shape-structure–activity relation

**DOI:** 10.1039/d1na90025k

**Published:** 2021-04-06

**Authors:** Ewelina Kuna, Dusan Mrdenovic, Martin Jönsson-Niedziółka, Piotr Pieta, Izabela S. Pieta

**Affiliations:** Institute of Physical Chemistry Polish Academy of Sciences 01-224 Warsaw Poland ipieta@ichf.edu.pl

## Abstract

Correction for ‘Bimetallic nanocatalysts supported on graphitic carbon nitride for sustainable energy development: the shape-structure–activity relation’ by Ewelina Kuna *et al.*, *Nanoscale Adv.*, 2021, **3**, 1342–1351, DOI: 10.1039/D0NA01063D.

The authors regret that the funding information in the Acknowledgements section of the original article was incorrectly shown.

The correct funding information is as follows:



The present research was financially supported by: (iii) the European Union’s Horizon 2020 research and innovation programme under the Marie Skłodowska-Curie grant no. 847413 and PMW programme of the Minister of Science and Higher Education in the years 2020–2024 no. 5005/H2020-MSCA-COFUND/2019/2.



The Royal Society of Chemistry apologises for these errors and any consequent inconvenience to authors and readers.

## Supplementary Material

